# Total Knee Arthroplasty in People with Hemophilia: Higher Incidence of Periprosthetic Joint Infection and 1-Year Revision/Re-Operation than the General Population and Lower Prosthetic Survival When Early Postoperative Bleeding Complications Occurred: Current Literature Review

**DOI:** 10.3390/jcm13082447

**Published:** 2024-04-22

**Authors:** Emerito Carlos Rodriguez-Merchan, Mario Mosconi, Hortensia De la Corte-Rodriguez, Eugenio Jannelli, Gianluigi Pasta

**Affiliations:** 1 Department of Orthopedic Surgery, La Paz University Hospital-IdiPaz, 28046 Madrid, Spain; 2 Osteoarticular Surgery Research, La Paz Hospital Institute for Health Research—IdiPAZ-La Paz University Hospital—Autonomous University of Madrid, 28046 Madrid, Spain; 3 Department of Clinical, Surgical, Diagnostic and Pediatric Sciences, University of Pavia, 27100 Pavia, Italy; mario.mosconi@unipv.it (M.M.); eugenio.jannelli@libero.it (E.J.); 4 Orthopedics and Traumatology Clinic, IRCCS Policlinico San Matteo Foundation, 27100 Pavia, Italy; gianluigipasta@yahoo.it; 5 Department of Physical and Rehabilitation Medicine, La Paz University Hospital-IdiPaz, 28046 Madrid, Spain; hortensiadelacorterodriguez@yahoo.es

**Keywords:** hemophilia, knee arthropathy, total knee arthroplasty, results, complications, prosthetic survival

## Abstract

The purpose of this narrative review of the recent literature is to analyze the outcomes, complications, and implant survival of total knee arthroplasty (TKA) carried out on people with hemophilia (PWH). It has been shown that TKA substantially alleviates preoperative pain and improves knee function and the patient’s quality of life. However, the complication rates of TKA range between 8.5% and 28.7, with postoperative hemarthrosis being the most frequent (7.6%). Besides, when comparing if the TKA was implanted before or after the year 2000, a reduction was found in the rates of periprosthetic joint infection—PJI (6.2% to 3.9%) and aseptic loosening (3.8% to 2.1%). Comparing prosthesis survival between PWH who had suffered early postoperative bleeding complications (EPBC) and patients who did not suffer EBPC, the mean survival duration was 17 years for the EPBC group and 22.1 years for the non-EPBC group. Survival rates were 80% for the EPBC group and 96.4% for the non-EPBC group. Compared to patients without hemophilia, PWH had a substantially higher incidence of PJI (Odds Ratio—OR 1.6) and 1-year revision/re-operation (OR 1.4). In short, although TKA substantially improves the quality of life of PWH, it is an intervention that has a non-negligible percentage of complications. TKA in PWH should preferably be performed only in highly specialized centers for the orthopedic treatment of hemophilia.

## 1. Introduction

Although the intricate molecular mechanisms underpinning the abnormal synovial hypertrophy in hemophilic arthropathy are still badly understood, some reports have demonstrated that a diversity of mediators might play a substantial role in blood-induced articular damage. Such mediators are thought to trigger a synovial over-reaction which, once started, might act independently of the intraarticular hemorrhage. Hemarthrosis causes intraarticular iron deposition, synovial proliferation and neo-angiogenesis, and cartilage and subchondral bone damage, triggering a vicious cycle that causes severe arthropathy. Albeit osseous damage may arise from a multifactorial process in people with hemophilia (PWH), articular hemorrhage seems to be an important contributor. This complex scenario eventually causes a clinical manifestation of hemophilic arthropathy. Spontaneous articular hemorrhage and recurrent hemarthroses cause hemophilic arthropathy—a debilitating condition which has a substantial negative impact on mobility and quality of life. Iron, cytokines, and angiogenic growth factors play a critical role in the beginning of the inflammatory process that affects the synovial tissue, articular cartilage, and subchondral bone, with early damages and molecular changes determining the maintenance of a chronic inflammatory condition. Synovitis is one of the earliest complications of intraarticular hemorrhage, and it is characterized by synovial hypertrophy, migration of inflammatory cells, and a high degree of neo-angiogenesis with subsequent bleeding. The pathogenic mechanisms and molecular pathways by which blood in the joints produces articular cartilage and subchondral bone destruction have yet to be fully comprehended. Both cytokines and matrix metalloproteinases and hydroxyl radicals may cause chondrocyte apoptosis. Members of the tumor necrosis factor receptor superfamily (such as the molecular triad: osteoprotegerin—OPG; receptor activator of nuclear factor kB—RANK; RANK ligand—RANKL) seem instead to play an important role in the inflammatory process. These pathogenic processes interact with each other and eventually cause a fibrotic articulation and the disabling condition typical of hemophilic arthropathy [1].

Primary hematological prophylaxis from infancy averts the start of hemophilic arthropathy. Hence, such prophylaxis should be commenced in childhood and continued throughout the life of PWH, as it is fundamental to the management of hemophilia [2,3,4,5].

When hematological treatment is insufficient or PWH do not have the monetary means to access care, and repetitive hemarthrosis and the subsequent chronic synovitis and degeneration of the articular cartilage cannot be averted, PWH will suffer from severe knee pain and substantial functional impairment of the knee. At that moment, in addition to an appropriate hematological management strategy, other therapeutic means—such as pain killers, anti-inflammatory medication (COXIBS), physical and rehabilitation medicine, and intraarticular injections of hyaluronic acid—have to be put into effect. If such means also are unsuccessful, total knee arthroplasty (TKA) should be contemplated as a last recourse [6,7,8,9].

It has been clearly shown that TKA in PWH suffering from severe arthropathy of that joint alleviates knee pain and improves knee function and quality of life. However, there are a number of data (related to complications and prosthesis duration) that are important to highlight so that PWH and the multidisciplinary team treating hemophilic patients have an adequate picture of what TKA really means today [10,11,12,13,14,15,16,17,18,19,20,21,22,23,24,25,26,27,28,29,30,31,32,33,34,35,36,37,38,39,40,41,42]. The purpose of this article is to analyze recent literature on the results of TKA in PWH, especially regarding its complications (with special attention to periprosthetic joint infection—PJI) and prosthetic survival.

In a search carried out in PubMed on 1 February 2024 using the keywords “total knee arthroplasty” and “hemophilia” from the start of the search machine (January 1996) to 3 April 2024, 301 articles were found. Of these, 48 were selected because they specifically presented data on the results of TKA in PWH, its complications (with special attention to PJI) and revision rates. One reference (number 1) was taken from a book published by the first author of this article.

## 2. Perioperative Hemostasis

It is well known that TKA have to be carried out in consultation with expert hematologists, who will initiate and oversee the intravenous (IV) infusion of concentrates of the deficient factor (factor VIII (FVIII) or factor IX (FIX)), either in recombinant or plasma-derived form, in the appropriate dosage (ideally for 10–14 days). In PWH with inhibitors, aPCCs (activated prothrombin complex concentrates) or rFVIIa (recombinant FVII activated) are usually utilized [43,44,45]. Besides, two important risk factors for significant perioperative blood loss have been found in the literature: clotting factor level < 93.5% and hematocrit level < 38.2% [36]. Table 1 summarizes the reported recommendations for peak factor levels and duration of factor replacement in TKA in PWH [46,47,48,49]. Table 2 shows the drugs available for perioperative hemostasis in PWH during TKA [45].

## 3. Surgical Approach

Orthopedic surgeons who are going to implant a TKA in PWH with severe knee arthropathy should use the standard approach when the preoperative range of motion (ROM) is over 35° and knee flexion is greater than 35°. Otherwise, they should carry out a V-Y quadricepsplasty [33]. Ono et al. demonstrated that the preoperative ROM and flexion were significantly associated with V-Y. The threshold values of preoperative flexion and ROM resulting in V-Y using receiver operating characteristic analysis were 45° and 35°, respectively.

## 4. Results and Complications

In 2019, Santos Silva et al. analyzed 18 TKA carried out in 15 PWH during a 24-year period. The mean follow-up was 11.3 years [30]. The ten-year survival rate with prosthesis removal as the end point was 94.3%. The complication rate was 27.8% (two infections, two postoperative stiff knees, and one recurrent hemarthrosis). The conclusion was that after adequate medical optimization and with prompt rehabilitation, TKA can be carried out in PWH with good clinical outcomes and survivor rates comparable to those of non-hemophilic patients.

In 2020, Oyarzun et al. analyzed 41 TKAs (19 cases were bilateral) [31]. Six individuals needed revision (6.66%) due to infection. The TKA survival at 5 years was 92% (range, 82–96%). This study supported the idea that TKA improves function and range of motion (ROM) in hemophilic knee arthropathy.

In a single-surgeon cohort study including 1515 individuals who experienced 2083 TKAs for osteoarthritis (OA), hemophilic arthropathy (HA), or rheumatoid arthritis (RA), Li et al. compared the results in 2020 [32]. The overall complication rate in the HA cohort was 21.8%, which was much higher than in the OA or RA group (7.1% and 8.7%, respectively). The dominant complications were prosthetic loosening and wound dehiscence. For HA, more complications occurred in the period more than 1 year after TKA when compared with OA (33.33% versus 11.43%). Among the potential risk factors, patients with hemophilia B and severe hemophilia had significantly higher complication rates. When compared with OA or RA, HA individuals had different characteristics related to complications, including the higher complication rate, different complication distribution and later occurring time. In HA individuals who experienced TKA, hemophilia B and severe hemophilia were risk factors of complications.

In 2020, Ono and Takedani analyzed 11 TKAs in PWH A who had no history of inhibitors. A pneumatic compression device was used from the beginning of the procedure until the individual could carry out standing exercises (day 2). Ultrasonography (US) of the lower extremities was performed prior to and after surgery (day 2) to detect DVT (deep vein thrombosis). Contrast-enhanced CT (computed tomography) was performed after surgery (day 7) to detect venous thromboembolism (VTE). D-dimer was measured pre- and postoperatively [33]. The mean age at the time of operation was 50.5 years. DVT was not encountered during either pre- or postoperative examinations by US, but contrast-enhanced CT detected DVT in two cases. No patients showed clinical signs of VTE during hospitalization, and no additional treatment for VTE was required. No episodes of unexpected bleeding were observed. Contrast-enhanced CT detected DVT in 18% of PWH A who experienced TKA despite no detection of DVT occurring via US.

In 2021, (Rosas et al.) PWH A and B were matched to controls using a 1:1 random matching process based on age, gender, Charlson Comorbidity Index (CCI), and select comorbidity burden [34]. A total of 4034 PWH were identified as having experienced TKA. About 44.8% were between the ages of 65 and 74 and 62.4% were female. Although the CCI was identical in both groups, individual comorbidities that were not controlled for varied substantially. Hemophilia was associated with higher odds of PJI (1.78 versus 0.98%). The 90-day reimbursements were higher for PWH (mean: USD 22,249 vs. USD 13,017). Medicare beneficiaries with a diagnosis of hemophilia experience more frequent postoperative complications and incur greater 90-day costs than matched controls after TKA.

In 2022, Chen et al. analyzed 103 primary TKAs carried out on 75 PWH [35]. Unilateral TKA was performed on 47 individuals and bilateral TKAs on the remaining 28 patients, including 12 simultaneous and 16 staged surgeries. The mean age at surgery was 32.3 years, and the mean follow-up duration was 77.9 months. Failure was found in eight individuals (8.5%) at a mean of 32.8 months after surgery. Four individuals suffered aseptic loosening, whereas infection occurred in four patients. The 10-year prosthesis survivorship was 88.6%. For individuals experiencing unilateral TKA, the mean length of hospital stay was 15 days. The mean cost of the factor supplement was USD 43,543 with a mean four-unit packed red blood cells transfusion. The total admission cost was USD 48,326. In terms of cost-effectiveness, bilateral simultaneous TKA was preferable to staged procedures.

A total of 92 TKA (78 hemophilia A and 14 hemophilia B) were carried out for hemophilic arthropathy (Kim et al., 2022) [36]. Perioperative blood loss (PBL) was calculated using the hemoglobin (Hb) balance method, and individuals were categorized into two groups: group H (higher blood loss than the mean PBL, n = 36) and group L (lower blood loss than the mean PBL, n = 56). The body mass index (BMI), operation day hemoglobin, hematocrit, and coagulation factor level (VIII or IX) were analyzed, including demographic and laboratory data. The mean PBL volume during TKA for hemophilic arthropathy of the knee was 542.3 mL. Lower hematocrit on the operation day and coagulation factor level were independent risk factors for increased PBL. Receiver-operating characteristic analysis identified these cutoff values for predicting increased PBL: operation day coagulation factor level 93.5% and hematocrit level of 38.2%. The PBL increased as hematocrit and coagulation factor levels diminished on the operation day. A coagulation factor level <93.5% or hematocrit level of <38.2% may be a significant risk factor for increasing PBL.

Shen et al. (2022) explored the definite factors affecting the hidden blood loss in TKA for 92 PWH [37]. The hidden blood loss of PWH experiencing TKA was 1069.51 mL, and the patient’s age was positively correlated with hidden blood loss, while tranexamic acid, FVIII prophylaxis, and incremental in vivo recovery were negatively correlated with hidden blood loss. Intraoperative intraarticular injection of tranexamic acid was advised to diminish hidden blood loss. FVIII prophylaxis was recommended for every individual.

Twenty-eight patients (32 knees) were included in the study of Wang et al. (2022) [38]. The follow-up was 69.1 months. The incidence of complications was 15.6%. The satisfaction was 100% in this mid-term study.

A retrospective study published in 2022 by Cambolat et al. included 82 patients who were hemophilic and non-hemophilic TKA patients operated on under general anesthesia [39]. A total of 73 individuals were assessed and divided into two cohorts according to the diagnosis of hemophilia: 36 patients were investigated in the hemophilic cohort and 37 patients in the non-hemophilic cohort. Postoperative tramadol consumption and pethidine consumption were substantially higher in the hemophilia cohort. The length of stay in the hospital was also substantially longer in the hemophilic cohort. Acute postoperative pain management of PWH should be planned as personalized, multimodal pre-emptive analgesia.

Goker et al. (2022) analyzed forty-five TKAs in 29 PWH [40]. This study assessed the effects of early major postoperative bleeding (MPOB) on the final functional result, complications, and implant survival of TKA. Ten patients (10 TKAs) (34%) experienced major bleeding during the postoperative period. Three of these patients had hemarthroses (10.2%), one patient had a hematoma (3.4%), one patient exhibited hemorrhagic bullae formation (3.4%), and five had excessive/prolonged bleeding from the wound (17%). The bleeding group (34%) had significantly worse Hospital for Special Surgery (HSS), Knee Society Score (KSS), and Knee Society Score Functional (KSS-F) scores compared with the controls. One of the patients with postoperative hemarthrosis also had a transient common peroneal nerve palsy and one patient (3.4%) had a periprosthetic fracture. Three knees (6.6%), two of whom were in the bleeding group, developed PJIs. Four knees (8.8%) in three patients underwent revision surgery, and two knees (4.4%) ended up in arthrodesis. The prosthetic survival was 17.04 years for the bleeding group and 22.15 years for the control group. Survival rates were 80% for the bleeding group and 96.4% for the control group. In this study, MPOB after TKA in PWH was common and led to substantially worse function. MPOB after TKA in PWH was associated with a higher rate of complications and lower survival rates, although the differences were not statistically significant. 

In the systematic review and meta-analysis published by Fenelon et al. (2022) a total of 1210 TKAs were carried out in 917 PWHs [41]. The mean age of patients was 38.5 years with a mean length of follow-up of 7.1 years. The total complication rate was 28.7%, with 19.3% needing a further procedure. HSS Knee Score improved by 44.6 points and KSS-F improved by 35.9 points. Total ROM improved by 22.3°. The most common complication was postoperative hemarthrosis (7.6%, 92 TKAs). Deep infection (6.2% versus 3.9%) and aseptic loosening (3.8% versus 2.1%) rates fell between period B (before the year 2000) and period A (after the year 2000). TKA in PWHs was a successful procedure improving function, alleviating pain, and improving ROM. PWHs underwent TKA at a younger age and had a higher risk of complications, though contemporary treatment had diminished these risks. PWHs can expect similar survivorship to the general population. 

A total of 26 PWH with 36 TKAs were followed up by Feng et al. (2023) for an average of 12.4 years [42]. Mild and enduring anterior knee pain was reported in seven knees (19%). Revision surgery was carried out in seven knees, with 10- and 15-year prosthesis survival rates of 85.8% and 75.7%, respectively. TKA was an efficacious procedure for individuals with end-stage hemophilic arthropathy, which alleviated pain, improved knee functions, reduced flexion contracture, and provided a high rate of satisfaction after more than 10 years of follow-up.

The aim of the study of Ono et al. (2023) was to optimize the surgical exposure in primary TKA for PWH and to propose a threshold angle of extension contracture in treating hemophilic knee joints, retrospectively [43]. Sixty-seven primary TKAs for PWH (mean age, 48 years) were performed, and incisional approaches to the joint included standard (58 cases) and V-Y quadricepsplasty (V-Y) (9 cases). The preoperative ROM and flexion were substantially associated with V-Y. The threshold values of preoperative flexion and ROM resulting in V-Y using receiver operating characteristic analysis were 45° and 35°, respectively. Primary TKA for PWH using a standard approach might be carried out before the stage preoperative flexion < 45° and ROM < 35°.

The aim of this retrospective study reported by Carulli et al. (2023) was to assess the long-run results and survival rates of TKA in a series of consecutive PWH affected by severe knee arthropathy [44]. These authors followed 65 PWH undergoing 91 TKAs, implanted using the same implant, which were characterized by an oxidized zirconium femoral component, coupled with a titanium tibial component and highly crosslinked polyethylene. The study showed a high survival rate of specific implants potentially linked to the choice of oxidized zirconium coupled with highly crosslinked polyethylene. These authors promoted the use of modern implants in PWH in order to ensure long-lasting positive outcomes.

A recent study (Challoumas et al., 2024) compared the results of TKA in PWH and matched controls [45]. PWH had a significantly higher incidence of the PJI and 1-year revision/re-operation. These data can be used by healthcare professionals counseling PWH who are considering a TKA as part of the informed consent process.

The possible complications of TKA in PWH should be well known to facilitate healthcare providers’ decision making and to help them avoid such complications as much as possible and treat them when they appear.

The complication rates published in the original articles are as follows: 27.8% (Santos Silva et al., 2019) [30]; 21.8% (Li et al., 2020) [32]; 8.5% (Chen et al., 2022) [35]; and 15.6% (Wang et al., 2022) [38]. In the systematic review and meta-analysis published by Fenelon in 2022, the complication rate was 28.7%, with 19.3% of PWH needing some type of further surgery [41]. In 2020, Oyarzun published a study in which the re-admission rate due to PJI was 6.6% [31].

**Figure 1:**
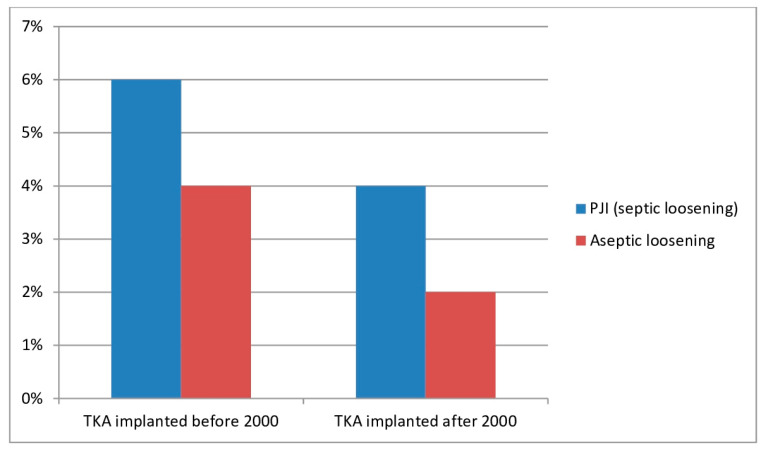
Rates of septic loosening due to periprosthetic joint infection (PJI) and of aseptic loosening after total knee arthroplasty (TKA) in people with hemophilia (PWH).

According to Fenelon et al., the most frequent postoperative complication after TKA was hemarthrosis (7.6%) [41]. In the same article, a comparison was made between TKAs implanted before 2000 and those implanted after 2000. It was found that the rates of PJI and aseptic loosening diminished: the rate of PJI diminished from 6.2% to 3.9% and the rate of aseptic loosening rate diminished from 3.8% to 2.1% (Figure 1) [41].

In 2023, Feng et al. published a paper claiming that 19% of PWH who underwent TKA suffered persistent pain of moderate intensity in the anterior part of their knees [42].

In the report of Ono and Takedani (2020), an 18% rate of DVT was found by means of contrast-enhanced CT. However, no cases of DVT were encountered by means of US [33]. In 2021, Rosas compared 90-day postoperative complications between PWH and people without hemophilia, revealing that the rate of PJI was higher in PWH [34].

In 2024, Challoumas et al. found that the odds of PJI after TKA in PWH were 1.6 times greater than the odds of PJI in PWH [45].

## 5. Prosthetic Survival 

**Figure 2:**
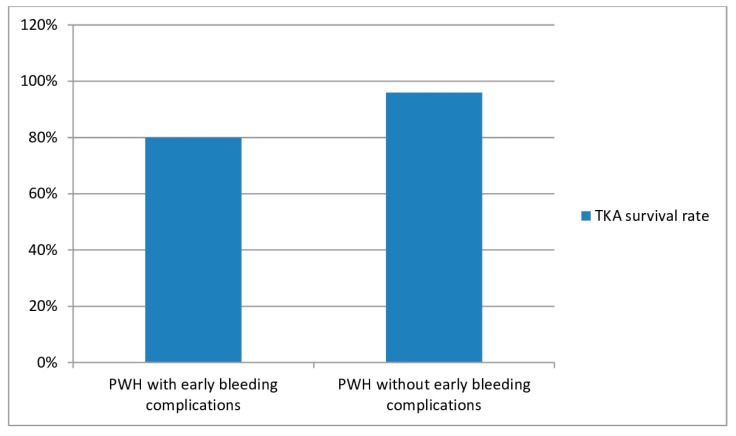
Rates of prosthetic survival of total knee arthroplasties (TKAs) implanted in people with hemophilia (PWH) who suffered early postoperative bleeding complications EPBC) versus those who did not suffer EPBC.

Regarding the rates of prosthetic survival, Oyarzun et al. (2022) observed a 5-year survival rate of 92% [30]. Santos Silva et al. (2019) encountered a 10-year survival rate, with prosthesis removal as the end point, of 94.3% [31]. In 2022, Chen et al. noted a 10-year survival rate of 88.6% [35]. In 2023, Feng et al. encountered a 10-year survival rate of 85.8% and a 15-year prosthesis survival rate of 75.7% [42]. In 2022, Goker et al. compared prosthesis survival between PWH who had suffered early postoperative bleeding complications—EPBC—(wound dehiscence, ecchymosis, intra-articular bleeding, hematoma, lengthy or repetitive bleeding episodes) after TKA and PWH who had not suffered EPBC. Kaplan–Meier analysis showed that the survival duration in the EPBC group was 17.04 years, while the survival duration in the non-EPBC was 22.15 years. Survival rates were 80% for PWH who suffered EPBC and 96.4% for PWH who did not suffer EPBC (Figure 2) [40].

Regarding the prosthetic design, Carulli et al. have recently stated that TKA in PWH has usually been carried out using cobalt–chrome femoral and titanium tibial components, coupled with standard polyethylene inserts [44]. However, Carulli et al. have shown a high survival rate (97.5% at a mean follow up of 12.3 years) with implants made of oxidized zirconium coupled with highly crosslinked polyethylene [44]. However, Carulli et al. did not compare implants made of oxidized zirconium coupled with a highly crosslinked polyethylene and titanium tibial component with cobalt–chrome femoral and titanium tibial components, coupled with standard polyethylene inserts (the design most frequently used in clinical practice). Therefore, their encouraging results will have to be confirmed in future comparative studies.

Table 3 summarizes all the information regarding the method, results, and conclusions of the 16 publications analyzed in this article [30,31,32,33,34,35,36,37,38,39,40,41,42,43,44,45].

In 2024, Challoumas et al. found that the odds of 1-year revision/re-operation after TKA in PWH were 1.4 times greater than the odds of 1-year revision/re-operation in people without hemophilia [45].

## 6. Discussion

TKA has to be carried out in consultation with expert hematologists that will prescribe and monitor the IV infusion of concentrates of the deficient factor (FVIII or FIX), either in recombinant or plasma-derived form, in the adequate dosage (ideally for 10–14 days). In PWH with inhibitors, aPCCs or rFVIIa are usually utilized [2,3,4,5,6,7,8,9,10,11,12,13,14,15,16,17,18,19,20,21]. 

In PWH, TKA improves the quality of life and offers good functional outcomes. TKA is safe, even in PWH with inhibitors. However, the risk of postoperative bleeding and infection of the surgical area is higher in PWH than in people without hemophilia. Consequently, the risk of a poor outcome after TKA is higher in PWH [30,31,32]. Therefore, TKA is associated with a non-negligible rate of complications. In other words, clinicians should consider TKA a major procedure associated with a high rate of complications. The fact that PWH present a higher incidence of PJI and 1-year revision/re-operation than the non-hemophiliac population could be due to insufficient hemostasis during the perioperative period, although it could also be related to a failure in the surgical technique. However, as far as we know, there are no data in the literature supporting this hypothesis.

It is therefore essential to ensure a good surgical technique is implemented and to control hemostasis well during the entire perioperative period.

The fact that PWH that suffer EPBC (wound dehiscence, ecchymosis, intra-articular bleeding, hematoma, and lengthy or repetitive bleeding episodes) have a lower prosthetic survival than PWH that do not suffer EPBC, in my opinion, could also be related to insufficient hemostasis during the perioperative period, but without forgetting that it could also be due to vascular injury sustained during surgery or due to insufficient surgical hemostasis. Therefore, good surgical hemostasis by the surgeon and good hemostasis control by the hematologist during the entire perioperative period are essential.

The fact that the rates of PJI and aseptic loosening of TKAs in PWH implanted after the year 2000 were lower those rates when the TKAs were implanted before the year 2000 can be explained by the improvements in prosthetic designs implemented after the year 2000.

## 7. Conclusions

TKA in PWH substantially alleviates preoperative pain and improves knee function and the patient’s quality of life. However, recent literature has shown that it is a surgical procedure that has a non-negligible rate of potential complications. Besides, TKA in PWH is associated with a higher incidence of PJI and 1-year revision/re-operation than in the general population and a lower prosthetic survival when early postoperative bleeding complications occurred. There has been no change in the treatment model of TKA in PWH. Besides, no consensus has been reached. The prosthetic designs are improving but the surgical technique is basically the same. TKA in PWH should be performed preferably only in highly specialized centers for the orthopedic treatment of hemophilia.

## 8. Limitations and Future Direction

The main limitations of this article are that it is a narrative review of the literature using only one search engine (PubMed) and that the selection of articles was based on the personal opinion of the authors. However, the publications on TKA in PWH make it clear that it is a safe technique that improves knee function and the quality of life of the patient who undergoes the procedure. It is essential that hematologists carry out thorough control of hemostasis and that orthopedic surgeons perform the operation in the correct way.

As a future direction, we believe that in the very near future, TKA in hemophilic patients will present results similar to those of TKA in patients with degenerative osteoarthritis.

## Figures and Tables

**Table 1:** Recommendations for peak factor levels and duration of factor replacement in total knee arthroplasty (TKA) in people with hemophilia (PWH).

	Hemophilia A	Hemophilia B
Preoperative peak factor activity objective	80–100%	60–80%
Postoperative peak factor activity objective	60–80% (days 1–3)	40–60% (days 1–3)
	40–60% (days 4–6)	30–50% (days 4–6)
	30–50% (days 7–14)	20–40% (days 7–14)

**Table 2:** Perioperative hemostasis in people with hemophilia (PWH) undergoing total knee arthroplasty (TKA).

Clotting Factor Concentrate (CFC)	Multiple CFC products are available for hemophilia A and B that can be used in the perioperative setting. The CFC product should be readily available at the site of surgery and postoperatively if needed, depending on the severity of the patient’s hemophilia and anticipated surgical risk. Most patients with hemophilia require CFC support in the perioperative setting. A preoperative bolus of CFC should be administered 30 to 60 min prior to the procedure. For patients without a history of an inhibitor, CFC dosing should be based on the patient’s weight, baseline factor levels, target factor level, and volume of distribution. Postoperative factor levels should be obtained depending on the half-life of the CFC: 8 to 12 h after the last dose of standard half-life FVIII CFCs and 12 to 24 h after the last dose of standard half-life FIX CFCs. If atypical or unexpected bleeding occurs, a stat factor level should be obtained to confirm sufficient levels, and alternative causes of bleeding should be considered, including anatomical drivers.
Bypassing Agents for Inhibitor Patients	Patients with FVIII or FIX inhibitors are typically treated with bypassing agents. The WFH recommends against the use of activated prothrombin complex concentrates (aPCCs; FVIII inhibitor bypass activity; FEIBA) for patients with congenital hemophilia B and inhibitors due to the risk of accumulation of clotting factors II, VII, and X, which can be associated with a higher risk of thrombotic complications. Rarely, patients with inhibitors may receive FVIII or FIX CFC when the inhibitor titer is negligible or low (<5 Bethesda units; BU), and CFC support is anticipated for a short duration with the understanding that reexposure to FVIII or FIX CFC results in an anamnestic response with a rise in the inhibitor titer and a loss of response to the CFC product.
Antifibrinolytics	The antifibrinolytics, tranexamic acid (TXA) and epsilon-aminocaproic acid, can be used as adjuvants for hemostatic support; these are particularly effective for mucosal bleeding. Overall, the use of TXA during major surgeries has not been shown to increase the risk of thromboembolism.

**Table 3:** Total knee arthroplasty (TKA) in people with hemophilia (PWH) from 2019 to 2024.

Authors [Reference]	Year	Methods	Results	Conclusions
Santos Silva et al. [30]	2019	Eighteen TKAs (15 PWH) during a 24-year period. Mean follow-up: 11.3 years. Mean age not available.	Ten-year survival rate with prosthesis removal as end point was 94.3%. Complication rate was 27.8% (two infections, two postoperative stiff knees, one instance of recurrent intra-articular bleeding).	TKA in PWH gave good clinical outcomes and survivor rates comparable to those of non-hemophilic patients.
Oyarzun et al. [31]	2020	41 TKA (19 cases were bilateral).	Six individuals needed revision (6.66%) due to infection. TKA survival at 5 years was 92%.	TKA improved function and ROM.
Li et al. [32]	2020	1515 individuals who experienced 2083 TKAs for osteoarthritis (OA), hemophilic arthropathy (HA), or rheumatoid arthritis (RA).	The overall complication rate in the HA cohort was 21.8%, which was much higher than the OA or RA group (7.1% and 8.7%, respectively).	Patients with hemophilia B and severe hemophilia had substantially higher complication rates.
Ono and Takedani [33]	2020	Eleven TKAs in PWH. Mean age: 50.5 years.	DVT was not encountered on either pre- or postoperative examinations by US, but contrast-enhanced CT detected DVT in two cases. No patients showed clinical signs of VTE during hospitalization.	Contrast-enhanced CT detected DVT in 18% of PWH A who experienced TKA despite no detection of DVT on US.
Rosas et al. [34]	2021	4034 TKAs in PWH A and B were matched to controls using a 1:1 random matching process based on age, gender, CCI, and select comorbidity burden.	Hemophilia was associated with higher odds of PJI (1.78 versus 0.98%).	PWH experienced more frequent postoperative complications than matched controls after TKA.
Chen et al. [35]	2022	103 primary TKAs (75 PWH). Unilateral TKA was performed on 47 individuals and bilateral TKAs on the remaining 28 patients, including 12 simultaneous and 16 staged surgeries. The mean age was 32.3 years, and the mean follow-up was 77.9 months.	Failure was found in 8 individuals (8.5%) at mean 32.8 months after surgery. Four individuals suffered aseptic loosening, whereas infection in 4. The 10-year prosthesis survivorship was 88.6%	In terms of cost-effectiveness, bilateral simultaneous TKA was preferable to staged procedures.
Kim et al. [36]	2022	A total of 92 TKA (78 hemophilia A and 14 hemophilia B). Perioperative blood loss (PBL) was calculated. Patients were categorized into two groups: group H (higher blood loss than the mean PBL, n = 36) and group L (lower blood loss than the mean PBL, n = 56).	The mean PBL volume during TKA for hemophilic arthropathy of the knee was 542.3 mL. Lower hematocrit on the operation day and coagulation factor level were independent risk factors for increased PBL.	A FVIII level < 93.5% or hematocrit level of <38.2% are significant risk factors for increasing PBL.
Shen et al. [37]	2022	This study explored factors affecting hidden blood loss (92 TKAs in PWH). Mean age not available.	The hidden blood loss was 1069.51 mL, and the age was positively correlated with the hidden blood loss. However, tranexamic acid, FVIII prophylaxis, and incremental in vivo recovery were negatively correlated with hidden blood loss.	Intraoperative intra-articular injection of tranexamic acid diminished hidden blood loss.
Wang et al. [38]	2022	Twenty-eight patients (32 TKAs). Mean follow-up: 69.1 months. Mean age not available.	The rate of complications was 15.6%.	The rate of satisfaction was 100%.
Cambolat et al. [39]	2022	73 TKAs (36 PWH and 37 patients without hemophilia). Mean age not available.	Postoperative tramadol consumption and pethidine consumption were substantially higher in PWH.	The length of stay in the hospital was also substantially longer in PWH.
Goker et al. [40]	2022	Forty-five TKAs in 29 PWH. This study assessed the effects of early major postoperative bleeding (MPOB) on the final functional result, complications, and implant survival of TKA. Mean age not available.	Ten patients (10 TKAs) experienced major bleeding during the postoperative period. Three of these patients had hemarthroses, one patient had a hematoma, one patient had hemorrhagic bullae formation, and five had excessive/prolonged bleeding from the wound. The bleeding group had significantly worse HSS, KSS, and KSS-F scores compared with controls.	In this study, MPOB after TKA in PWH was common and led to substantially worse function. MPOB after TKA in PWH was associated with a higher rate of complications and lower survival rates, although the differences were not statistically significant. Prosthetic survival was 17.04 years for the bleeding group and 22.15 years for the control group. Survival rates were 80% for the bleeding group and 96.4% for the control group.
Fenelon et al. [41]	2022	Systematic review and meta-analysis. A total of 1210 TKAs were carried out in 917 PWHs. The mean age of patients was 38.5 years with a mean length of follow-up of 7.1 years.	The complication rate was 28.7%, with 19.3% needing a further procedure. The HSS Knee Score improved by 44.6 points and KSS-F improved by 35.9 points. Total ROM improved by 22.3°. The most common complication was postoperative hemarthrosis (7.6%, 92 TKAs). PJI (6.2% versus 3.9%) and aseptic loosening (3.8% versus 2.1%) rates fell between period B (before the year 2000) and period A (after the year 2000).	TKA in PWHs is a successful procedure, improving function, alleviating pain, and improving ROM. PWHs underwent TKA at a younger age and had a higher risk of complications, though contemporary treatment diminished these risks.
Feng et al. [42]	2023	Twenty-six PWH with 36 TKAs were followed up for an average of 12.4 years. Mean age not available.	Mild and enduring anterior knee pain was reported in 7 knees (19%). Revision surgery was carried out in 7 knees, with 10- and 15-year prosthesis survival rates of 85.8% and 75.7%, respectively.	TKA alleviated pain, improved knee functions, reduced flexion contracture, and provided a high rate of satisfaction after more than 10 years of follow-up.
Ono et al. [43]	2023	This study proposed a threshold angle of extension contracture in treating hemophilic knee joints, retrospectively. Mean age 48 years.	Sixty-seven primary TKAs for PWH (mean age, 48 years) were performed, and incisional approaches to the joint included standard (58 cases) and V-Y quadricepsplasty (V-Y) (9 cases). The preoperative ROM and flexion were substantially associated with V-Y.	Primary TKA for PWH using a standard approach might be carried out before the stage preoperative flexion < 45° and ROM < 35°.
Carulli et al. [44]	2023	This study assessed the long-run results and survival rates of TKA in a series of consecutive PWH affected by severe knee arthropathy. Mean age not available.	These authors followed 65 PWH undergoing 91 TKAs, implanted using the same implant, characterized by an oxidized zirconium femoral component, coupled with a titanium tibial component and highly crosslinked polyethylene.	The study showed a high survival rate: 97.5% at a mean follow-up of 12.3 years.
Challoumas et al. [45]	2024	This study compared the results of TKA in PWH versus matched controls. Mean age not available.	PWH had a significantly higher incidence of PJI and 1-year revision/re-operation.	The odds of 1-year revision/re-operation after TKA in PWH were 1.4 times greater than the odds of 1-year revision/re-operation in people without hemophilia.

ROM = range of motion; DVT = deep vein thrombosis; CT = computed tomography; VTE = venous thromboembolism; US = ultrasonography; CCI = Charlson Comorbidity Index; PJI = Periprosthetic joint infection; HSS = Hospital for Special Surgery; KSS = Knee Society Score; KSS-F = Knee Society Score Functional.
